# Ranpirnase (OKG-0301), a Novel Ribonuclease, Demonstrates Antiviral Activity against Adenovirus in the Ad5/NZW Rabbit Ocular Replication Model

**DOI:** 10.3390/pathogens11121485

**Published:** 2022-12-07

**Authors:** Eric G. Romanowski, Kathleen A. Yates, Eric J. Daniels, Brian M. Strem, John E. Romanowski, Regis P. Kowalski

**Affiliations:** 1 The Charles T. Campbell Ophthalmic Microbiology Laboratory, UPMC Eye Center, Department of Ophthalmology, University of Pittsburgh School of Medicine, Pittsburgh, PA 15213, USA; 2 Okogen Pty Ltd., Melbourne, VIC 3000, Australia

**Keywords:** ranpirnase, RNase, antiviral, eye, adenovirus, EKC, animal model

## Abstract

Adenovirus ocular infections are common ocular viral infections seen worldwide, for which there is no approved antiviral therapy available. Ranpirnase is a novel ribonuclease which preferentially degrades tRNA resulting in an inhibition of protein synthesis. The study goal was to determine the anti-adenoviral activity of topical formulations of ranpirnase (OKG-0301) on adenoviral replication in the Ad5/NZW rabbit ocular replication model. NZW rabbits were inoculated in both eyes with human adenovirus type 5 (HAdV5) after corneal scarification. A day later, topical therapy was initiated in both eyes with 0.03% OKG-0301, 0.003% OKG-0301, saline or 0.5% cidofovir. Eyes were cultured to determine HAdV5 eye titers over 2 weeks. OKG-0301 (0.03% and 0.003%) and 0.5% cidofovir decreased viral titers compared to saline. Furthermore, both OKG-0301 formulations and 0.5% cidofovir shortened the duration of the HAdV5 infection compared to saline. Both 0.03% OKG-0301 and 0.003% OKG-0301 demonstrated increased antiviral activity compared to saline in the Ad5/NZW rabbit ocular replication model. The antiviral activity of the OKG-0301 groups was similar to that of the positive antiviral control, 0.5% cidofovir. Ranpirnase (OKG-0301) may be a potential candidate for a topical antiviral for adenoviral eye infections. Further clinical development is warranted.

## 1. Introduction

**Figure 1:**
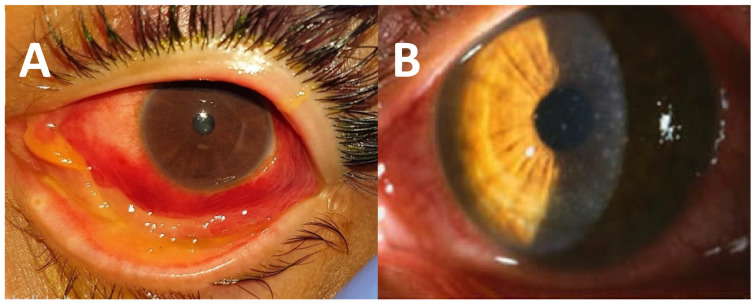
These photographs present (**A**) a patient with EKC and a pseudomembrane and (**B**) a patient with subepithelial corneal infiltrates. Photograph A courtesy of the Campbell Lab and Photograph B courtesy of Francis S. Mah, MD.

Human adenoviruses (HAdV) are the etiological agents of the most common ocular viral infections worldwide, accounting for up to 75% of all conjunctivitis cases [1]. Approximately 1 million cases of adenoviral conjunctivitis are seen annually in Japan [1]. These ocular infections manifest in three major ocular clinical syndromes. The most severe is epidemic keratoconjunctivitis (EKC) which involves the conjunctiva as well as the cornea [1]. The ophthalmic signs are mostly bulbar conjunctival redness, conjunctival chemosis, tarsal follicular response, petechiae, or possibly subconjunctival hemorrhage and pseudomembrane [1] (Figure 1A). Symptoms of EKC can last from 7 to 21 days with the patient remaining infectious for 10–14 days [1]. This infection is highly contagious, and outbreaks have been seen in healthcare facilities and nursing homes [1].

Pharyngeal conjunctival fever is a less severe form of adenoviral ocular infections that is seen mostly in children [1]. This syndrome is characterized by conjunctivitis, sore throat, and preauricular lymph node enlargement [1]. Follicular conjunctivitis is a least severe syndrome which is characterized by conjunctivitis without a sore or preauricular lymph node enlargement [1]. These acute infections can cause significant lost time from school and work. Two weeks is the typical isolation period for these patients. In the long term, these infections can produce vision-altering subepithelial corneal infiltrates that can last from months to years (Figure 1B). The infiltrates lead to topical corticosteroid treatment, which will temporarily resolve the infiltrates. However, tapering the corticosteroids can lead to the reappearance of the infiltrates. Long-term corticosteroid treatment can produce adverse effects such as glaucoma, cataracts, and superinfection.

Currently, there is no approved antiviral for the treatment of these infections. An effective antiviral agent for adenoviral ocular infections would fulfill an unmet medical need in ophthalmology. It would reduce patient pain and suffering during the acute infection and by decreasing the amount of virus on the eye, the formation of the vision-altering subepithelial infiltrates can be reduced or eliminated.

Several antiviral categories and strategies have been evaluated as potential treatments of HAdV eye infections. Nucleoside analogs [2,3,4,5,6], direct-inactivating, antiseptic-type agents [7,8,9], immunoglobulins [10], and dendrimers [11] have all been evaluated as antiviral agents for these infections. None of these antiviral agents have thus far been approved for use.

A new approach to antiviral development is the use of ribonucleases, enzymes that degrade RNA. Ranpirnase is a ribonuclease that is extracted from the oocytes of Northern leopard frogs *Rana pipiens*, (also known as *Lithobates pipiens*) which belongs to the RNase A superfamily [12]. Ranpirnase was originally developed as an anticancer therapy (Pannon, P-30, and Onconase for Injection [TMR-004]) but is now being repurposed as an antiviral agent [13].

Ranpirnase is a small enzyme (11.82 kDa). Its active site contains the catalytic triad (His10, Lys31, and His97) that is characteristic of the RNase A superfamily [12]. However, ranpirnase prefers to cleave the phosphodiesterase bond on the 5’ side of guanine in contrast to RNase A which prefers to cleave adenine at this site [12].

Ranpirnase has been extensively studied for its antitumor activity for which it has a unique mechanism of action. It is a biological drug that is administered extracellularly but acts intracellularly; therefore, it must be internalized into the cell [12]. Ranpirnase, a highly cationic enzyme, binds to the negatively charged cell surface via the cell-surface receptor, heparin sulfate. Ranpirnase then forms an equilibrium complex with heparin sulfate [12]. After forming the cell-surface complex, ranpirnase is internalized through endocytosis and is routed to endosomes [12]. Once in the cytosol, ranpirnase resists degradation by the ribonuclease inhibitor protein due to its extraordinary conformational stability [12]. Within the cell, transfer RNA (tRNA) is the main target of ranpirnase leaving ribosomal RNA (rRNA) and messenger RNA (mRNA) largely intact. This is most likely due to proteins bound to rRNA and mRNA which protect them from ranpirnase cleavage [12]. The degradation of tRNA inhibits protein synthesis and leads to the arrest of the cell cycle [13].

In contrast, much less is known about its mechanism of action as an antiviral. It has been speculated that its antiviral mechanism of action is similar to its anti-tumor activity in that the RNA interference results in direct virus inhibition, degradation of viral RNA, disruption of TNF-α signaling, and changes in host-cell gene expression [13,14]. Ranpirnase has been shown to have antiviral activity against rabies viruses [13], HIV [15,16], HPV [17], and Ebola virus [18].

It has been speculated that ranpirnase may have antiviral activity against adenovirus infections. This prompted this study where the goal was to evaluate the anti-adenovirus activity of topical ocular formulations of ranpirnase, heretofore known as OKG-0301, in the Ad5/NZW rabbit ocular replication model. We have used this model to evaluate the effect of several antivirals on adenovirus replication in vivo [2,3,4,5,7,10,11].

## 2. Materials and Methods

### 2.1. Experimental Drugs

0.03% and 0.003% OKG-0301 were prepared in intravenous saline from 1 mg vials of ranpirnase that were provided by Okogen, Pty Ltd., Melbourne, VIC, AUS. Cidofovir 0.5% (CDV) was diluted in intravenous saline from the 7.5% injectable form of cidofovir (Cidofovir Injection, [Heritage Pharmaceuticals Inc., Eatontown, NJ, USA]) and functioned as the positive antiviral control. Intravenous saline (0.9% Sodium Chloride Injection USP [Baxter Healthcare Corp. Deerfield, IL, USA]) functioned as the diluent for the experimental antivirals and as the negative control (CON).

### 2.2. Adenovirus Strain and Cells

The ocular HAdV5 strain used in this study was recovered from a patient with typical adenoviral ocular disease at the Charles T. Campbell Ophthalmic Microbiology Laboratory at the UPMC Eye Center, Department of Ophthalmology, University of Pittsburgh School of Medicine. It was determined to be adenovirus type 5 (HAdV5) using a standard serum neutralization technique [19]. Briefly, 100 TCID_50_ in 0.1 mL of the virus isolate was separately mixed for 1 h with 0.1 mL of several dilutions (16, 8, 4, and 2 Inhibitory Units/mL) of antisera of several adenovirus types purchased from ATCC (American Type Culture Collection, Manassas, VA, USA). After incubation, the virus-antibody samples were inoculated onto separate wells of a 96-well plate containing A549 human lung carcinoma cells (American Type Culture Collection, Manassas, VA, USA). The plates were incubated at 37 °C, in 5% CO_2_ for up to 10 days. Cells that were treated with the virus-antibody mixture that did not demonstrate cytopathic effect (CPE) denoted to which adenovirus type the isolate belonged.

The use of this de-identified isolate did not require an Institution Review Board (IRB)/Ethics committee approval because the patient contact or personal information was not compromised.

A549 human lung carcinoma cells (RRID:CVCL_0023) (ATCC) were used in this study.

The stock of the HAdV5 isolate was prepared in A549 cells. Briefly, the cells were grown to 85–95% confluence in tissue culture media consisting of 1X Minimum Essential Medium (Gibco/ThermoFisher Scientific, Waltham, MA, USA) with 1% Gentamicin Solution (50 mg/mL) (Sigma-Aldrich, St. Louis, MO, USA), 10% Penicillin-Streptomycin (5000 U/mL) (Gibco), 25 µg/mL Amphotericin B (Sigma-Aldrich), and 10% Fetal Bovine Serum (Corning, Glendale, AZ, USA). The media were removed from the A549 cells, then the cells were inoculated with a small volume of HAdV5 at a multiplicity of infection (MOI) of 5 to 10. Following a 3-h adsorption period, the inoculum was removed, and media were added to the cells. The cells were incubated at 37 °C, in 5% CO_2_. When the cells exhibited 100% CPE (1–3 days), the cells were scraped from the flask and the media plus cells were transferred to a 50 mL polypropylene conical tube. The cells were centrifuged for 5 min at 1800 RPM at 4 °C. The supernatant was discarded. The pellet was resuspended in media. Three cycles of freeze/thaw/vortex are preformed using liquid N_2_ and a 37 °C water bath. The virus was then aliquoted and stored in a −80 °C freezer until use. The titer of the HAdV5 stock was determined as described in Section 2.5.

HAdV5 was used in this study, as it was in previous ocular adenovirus antiviral studies [2,3,4,5,7,10,11]. It has been shown that human Species C adenoviruses (HAdV1, HAdV2, HAdV5, and HAdV6) have the ability to replicate in the corneas of NZW rabbits whereas the more common adenovirus strains seen in EKC patients, HAdV8 and HAdV19 (which is now designated as HAdV64 [20]) do not [21]. Therefore, we chose to use HAdV5 as the representative adenovirus to be used in our rabbit studies.

### 2.3. Experimental Animals

New Zealand White female rabbits weighing 1.1–1.4 kg (approximately 6 weeks old) were obtained from the Oakwood Research Facility through Charles River Laboratories, Wilmington, MA. This study conformed to the ARVO Statement on the Use of Animals in Ophthalmic and Vision Research. University of Pittsburgh Institutional Animal Care and Use Committee (IACUC) approval (Protocol #16027750) was granted, and US Animal Welfare Act guidelines were observed. The authors confirm that this study complied with the ARRIVE guidelines.

### 2.4. Experimental Design

This experiment was carried out using duplicate trials resulting in a total of a total of 38 rabbits. After general anesthesia with intramuscular injections of ketamine (40 mg/kg) and xylazine (4 mg/kg) was achieved, topical anesthesia was produced with 2 drops of 0.5% proparacaine. The rabbits were the transferred to a table fitted with an operating microscope where, 12 cross-hatched strokes of a #25 sterile needle were performed on both corneas. The rabbits were then inoculated with 50 µL (1.5 × 10^6^ PFU/eye) of HAdV5 in both eyes. Rabbits were treated with intramuscular injections of ketoprofen, 1.5 mg/kg on the day of inoculation and the next day. The following morning, the rabbits were randomly allocated to one of four topical treatment groups: (1) 0.03% OKG-0301—8X/day for 9 days (n = 9); (2) 0.003% OKG-0301—8X/day for 9 days (n = 10); (3) Saline—8X/day for 9 days (n = 10); and 0.5% CDV—2X/day for 7 days (n = 9). Rabbits were topically treated in both eyes according to the regimens described above. Cidofovir was administered only twice daily for 7 days as this is our standard treatment regimen using this agent [3,4,5,7,10,11]. Following induction of ocular anesthesia with 0.5% proparacaine, the ocular surfaces were swabbed to recover HAdV5 from the tear film and corneal and conjunctival surfaces on days 0, 1, 3, 4, 5, 7, 9, 11, and 14 post-inoculation at least 1 h after the final antiviral dose on those days when administered. The swabs were inserted into snap-cap tubes containing 1 mL of the tissue culture media described above and were frozen at −80 °C until the plaque assays were performed. The rabbits were euthanized following the final culture with an overdose of intravenous Euthasol solution following systemic anesthesia with ketamine and xylazine as described above.

The number of animals per group (n = 10) was based on previous studies [3,4,5,7,10]. One animal each was lost from the 0.03% OKG-0301 and the CDV groups during the study. The treatment regimen (8 times per day) was based on the maximum number of doses that can be given in a normal workday. The rabbits in each experimental group were randomly allocated upon arrival and pen-housed together to provide social housing and to avoid any potential errors in topical antiviral treatments.

### 2.5. Plaque Assay to Determine the Ocular HAdV5 Titers

The eye cultures to be quantified were thawed, diluted, and 0.1 mL of the undiluted and diluted samples were inoculated in duplicate onto A549 cell monolayers contained in 24-well multiplates. The viral inoculum was adsorbed for 3 h after which 1 mL of tissue culture media containing 0.5% methylcellulose was added to the wells. After 7 days of incubation at 37 °C in 5% CO_2_, the media were aspirated from the wells and the cells were stained with 0.5% gentian violet in formalin. The plates were dried and the numbers of HAdV5 plaques per well were counted using a dissecting microscope. The viral titers for each sample were calculated and presented as plaque forming units per milliliter (PFU/mL).

### 2.6. Statistical Analysis

The HAdV5 titer data were analyzed using Kruskal–Wallis ANOVA with Duncan’s multiple comparisons and Fisher’s Exact Test (True Epistat, Richardson, TX, USA). Significance was defined as *p* ≤ 0.05.

## 3. Results

### The Effect of Topical OKG-0301 on HAdV5 Replication in the Ad5/NZW Rabbit Ocular Replication Model

This antiviral study was conducted using the Ad5/NZW rabbit ocular replication model. This model evaluates ocular HAdV5 replication only. Signs of infection in the eyes are not produced in this model and therefore were not assessed. Ocular toxicity also was not evaluated in this model because of the corneal scarifications during the viral inoculations and the slight ocular trauma induced by the act of culturing the eyes. This is not a true representation of ocular toxicity that would be produced in naïve rabbit eyes. The ocular HAdV5 titer data were analyzed to determine the percentage of HAdV5 positive cultures per total for each culture day, the HAdV5 titers for each culture day, and the duration of shedding which is the length of the HAdV5 infection in the eyes.

**Figure 2:**
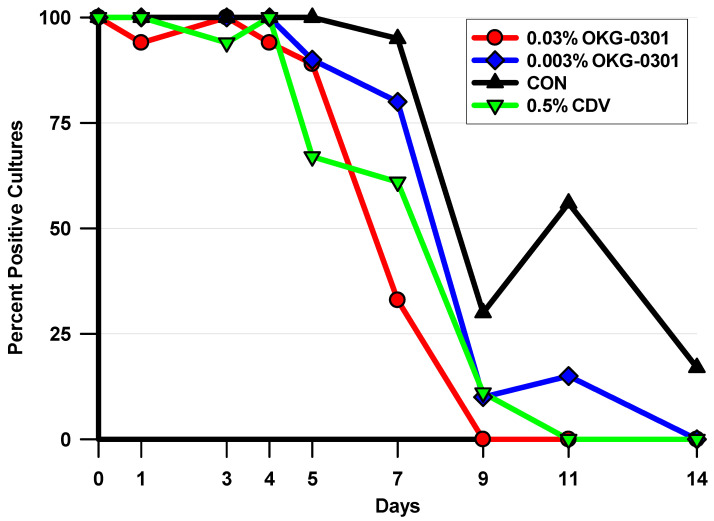
This figure presents the percentages of HAdV5 Positive Cultures per Total for each treatment group and culture day. These data represent the most rigorous of the viral outcome measures. Any sample with a detectable HAdV5 titer was considered to be a positive culture. Significant differences in the percentages of HAdV5 Positive Cultures per Total were demonstrated on the following days: **Day 5:** (CDV < CON); **Day 7:** (0.03% OKG-0301 = CDV < CON; 0.03% OKG-0301 < 0.003% OKG-0301); **Day 9:** (0.03% OKG-0301 < CON); **Day 11:** (0.03% OKG-0301 = CDV = 0.003% OKG-0301 < CON) (*p* ≤ 0.0208, FET). (<Indicates Significantly Fewer HAdV5 Positive Cultures per Total).

The most rigorous of the viral outcome measures is the percentage of HAdV5 positive cultures per total for each day that was assessed. Any sample with a detectable HAdV5 titer was deemed to be an HAdV5 positive culture. These data were analyzed using the Fisher Exact Test (FET) (Figure 2). Treatment with 0.03% OKG-0301 significantly decreased the percentage of HAdV5 positive cultures per total cultures on Days 7, 9, and 11 compared to CON (*p* ≤ 0.0208), while 0.003% OKG-0301 significantly decreased the percentage of HAdV5 positive cultures per total cultures compared to CON on Day 11 only (*p* = 0.0156). The positive antiviral control, 0.5% CDV significantly decreased the percentage of HAdV5 positive cultures per total cultures on Days 5, 7, and 11 compared to CON (*p* ≤ 0.0208). In addition, treatment with 0.03% OKG-0301 significantly decreased the percentage of HAdV5 positive cultures per total cultures on Day 7 compared to treatment with 0.003% OKG-0301 (*p* = 0.0076).

**Figure 3:**
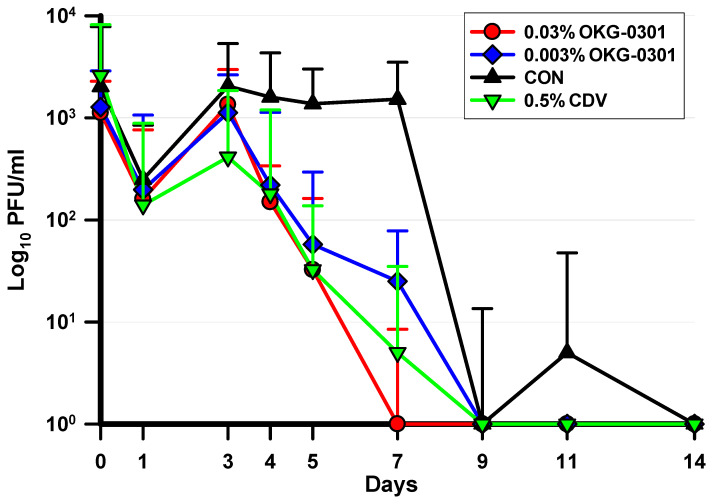
This figure presents the Log_10_ median ± interquartile ranges of HAdV5 ocular titers for each treatment group and culture day. Significant differences were produced on the following days: **Day 3:** (CDV = 0.003% OKG-0301 = 0.03% OKG-0301 < CON); **Day 4:** (0.03% OKG-0301 = CDV = 0.003% OKG-0301 < CON); **Day 5:** (CDV = 0.03% OKG-0301 = 0.003% OKG-0301 < CON); **Day 7:** (0.03% OKG-0301 < CDV < 0.003% OKG-0301 < CON); **Day 11:** (0.03% OKG-0301 = CDV = 0.003% OKG-0301 < CON) (*p* ≤ 0.05, K-W). (<Indicates Significantly Lower HAdV5 Ocular Titers).

The ocular HAdV5 titer data for each treatment group were analyzed using the Kruskal–Wallis ANOVA with Duncan’s multiple comparisons test (K-W). The data are expressed as Log_10_ median ± interquartile ranges (Figure 3). All antiviral groups demonstrated significantly lower HAdV5 titers compared to the saline control on Days 3, 4, 5, 7, and 11. There were no differences in HAdV5 titers among the antiviral groups on Days 3, 4, 5, and 11. However, on Day 7, the 0.03% OKG-0301 group significantly decreased the HAdV5 titers compared to the 0.5% CDV group, which in turn demonstrated significantly lower titers than the 0.003% OKG-0301 group. There were no significant differences among the groups on any of the other days.

The final days on which eyes had positive HAdV5 cultures were used to determine the duration of HAdV5 shedding. This viral outcome measure is shown as the median (±interquartile ranges) length of the infection in the eyes. Treatment with 0.03% OKG-0301 (5.0 ± 2.00 days), 0.003% OKG-0301 (7.0 ± 0.00 days), and 0.5% CDV (7.0 ± 2.75 days) significantly decreased the length of infection compared to the saline control (11.0 ± 4.00 days) (*p* ≤ 0.05, K-W). In addition, 0.03% OKG-0301 and 0.5% CDV significantly shortened the duration of HAdV5 shedding compared to 0.003% OKG-0301 (*p* ≤ 0.05, K-W). There was no significant difference between 0.5% CDV and 0.03% OKG-0301.

## 4. Discussion

Ribonucleases (RNases) are enzymes that cleave RNA. These enzymes play a key role in the development of mRNA and noncoding RNA, the operation of RNA interference, and the control of gene expression in all organisms [22]. A number of RNases have been shown to possess antiviral activity. RNase A was shown in 1976 to be an effective treatment for tick-borne encephalitis [23]. A form of RNase A isolated from the bovine pancreas has been approved in Russia for the treatment of viral meningitis and tick-borne encephalitis [23]. RNase L has been shown to trigger the transcription of the antiviral interferon-β gene [22]. Eosinophil-associated RNases produce antiviral activity by their capacity to cleave ssRNA [22]. The bacterial RNase, binase, has been shown to have in vitro antiviral activity against influenza A virus [24]. Another RNAse that has been extensively evaluated for antiviral activity is the amphibian RNAse, ranpirnase.

Ranpirnase has demonstrated antiviral activity against rabies viruses [13], HIV [15,16], HPV [17], and Ebola virus [18]. Its mechanism of antiviral action is specifically thought to involve the degradation of tRNA, but not mRNA and rRNA protected with proteins [22,25]. This hastens the tRNA degradation/synthesis cycle and degradation products that function as key protein translational nucleotides required for viral replication [22,25].

The demonstration of antiviral activity for ranpirnase against both RNA and DNA viruses led us to the current study for which we determined the antiviral activity of topical formulations of ranpirnase in an experimental model of an ocular adenovirus infection. We have used this model to evaluate a number of potential antiviral agents for the treatment of adenovirus ocular infections [2,3,4,5,7,10,11]. In this initial experimental study using an ocular formulation of ranpirnase, OKG-0301, we wished to maximize its potential antiviral efficacy and determine whether its antiviral activity was concentration dependent. Therefore, we topically treated the eyes 8 times per day for 9 days to maximize the exposure of the infected eyes to two concentrations of OKG-0301 (0.03% and 0.003%).

In the current study, both 0.03% and 0.003% OKG-0301 demonstrated antiviral activity against adenovirus in the Ad5/NZW rabbit ocular replication model. Both OKG-0301 formulations produced significant decreases in the percentage of HAdV5 positive cultures (0.03% OKG-0301 on Days 7, 9, and 11; 0.003% OKG-0301 on Day 11 only), decreases in daily HAdV5 ocular titers (Days 3, 4, 5, 7, and 11), and in the median duration of ocular HAdV5 shedding compared to the negative control (saline). These results are noteworthy because decreasing the adenovirus ocular titers and shortening the length of the infection can lead to a decrease in the morbidity associated with these infection as well as a possible reduction or prevention of the formation of vision altering subepithelial corneal infiltrates that are characteristic of adenovirus eye infections.

There were several concentration-dependent differences in viral outcome measures between the two OKG-0301 concentrations. The 0.03% OKG-0301 concentration significantly reduced the median duration of shedding by 2 days compared with 0.003% OKG-0301 and reduced the percentage of HAdV5 cultures and titers on Day 7.

To summarize this initial experimental study, two concentrations of an ocular formulation of ranpirnase, OKG-0301, demonstrated significant antiviral activity compared with saline in the Ad5/NZW rabbit ocular replication model. The antiviral efficacy demonstrated by the higher ranpirnase concentration was comparable to the positive antiviral control, 0.5% cidofovir, while the lower concentration was slightly less efficacious. There was a difference in antiviral efficacy between the two ranpirnase concentrations suggesting concentration-dependent antiviral activity.

As mentioned above, the goal of the current study was to only evaluate the antiviral activity of topical ranpirnase. The ocular toxicity of topical ranpirnase was not evaluated in this study. Subsequent to this experimental study, a Phase 2 clinical trial in patients with adenoviral conjunctivitis was initiated (NCT03856645) [26]. Preliminary data from an Association for Research in Vision and Ophthalmology (ARVO) abstract detail an interim analysis of the trial data which has suggested that OKG-0301 0.03% and 0.012% possesses concentration-dependent antiviral efficacy in patients with acute adenoviral conjunctivitis [26]. The interim data suggest that both concentrations reduced adenoviral titers 7 days after the onset of treatment [26]. Furthermore, both concentrations were deemed to be safe as no Treatment Emergent Serious Adverse Events (TESAEs) were seen [26].

## 5. Conclusions

In conclusion, ranpirnase demonstrated antiviral activity against an experimental ocular adenovirus infection in the Ad5/NZW rabbit ocular replication model. Based on the results of this study, further development of ranpirnase as an antiviral for the treatment of adenovirus ocular infections in patients is indicated.

## Data Availability

The data reported in this study are available in this manuscript.
